# Effect of Transcodent painless needles on injection pain in maxillary anterior infiltration: a split‐mouth controlled randomized clinical trial

**DOI:** 10.1002/cre2.475

**Published:** 2021-09-01

**Authors:** Farnoosh Razmara, Arjang Baghi, Farzaneh Afkhami

**Affiliations:** ^1^ Department of Oral and Maxillofacial Surgery, School of Dentistry Tehran University of Medical Sciences Tehran Iran; ^2^ Private Practice Tehran Iran; ^3^ Department of Endodontics, School of Dentistry Tehran University of Medical Sciences, International Campus Tehran Iran

**Keywords:** local anesthesia, needle, pain management, pain perception, supraperiosteal injections

## Abstract

**Objectives:**

The present study aimed to determine the pain perceived during supraperiosteal (infiltration) injection in anterior maxillary region by Transcodent painless needle tips in comparison to the regular needle tips.

**Material and methods:**

In this split‐mouth controlled randomised clinical trial, 30 patients were selected as candidates for cosmetic treatment who needed infiltration injections on both sides of canine area. They were each administered 0.9 mL of Lidocaine HCl 2% with epinephrine 1:100,000 in the buccal vestibules using two types of needle tips, Transcodent painless needle tip or regular needle tip. Immediately after the injection, the pain was measured using a 100 mm visual analog scale. The level of pain was statistically analyzed in the two groups using the parametric paired *t*‐test. A 5% significance level was considered for statically significant difference between two means.

**Results:**

In accordance with the results, the patients' level of pain were estimated as 18.3 ± 10.7 mm with Transcodent painless needle tips and 43.1 ± 13.1 mm in regular needle tip (*p* < 0.05).

**Conclusion:**

The Transcodent painless needle showed considerable reduction of pain in the anterior maxillary infiltration when compared to the regular needle tips.

## 1 INTRODUCTION

Dental anxiety describes the fear or stress that people have in visiting a dental unit for treatment. It is usually associated with a scare of needles, drills, pain, or other settings of dental practices. In addition to delaying and even avoiding dental treatment, dental anxiety may cause unexpected behaviors of patients that prevents the dentist from carrying out routine dental procedures. Anxiety in the patients and its repeated experience during dental visits also affect the dentists' efficiency and self‐confidence. Since providing effective dental services is instrumental in determining the health index of the society, anxiety in patients and their reluctance to receiving dental health services have a negative impact on the health index of the society (Afkhami et al., 2021; Amanat, 2004; Dau et al., 2017; Steele et al., 2013).

The provision of dental treatments almost certainly requires the use of local anesthesia to temporarily block the transmission of nerve impulses and abolish pain sensation. Dental operations and injections of local anesthesia are however among the most terrifying procedures for the patients. Pain management is among the main factors that affect the success of dental treatment and patient's satisfaction (Dau et al., 2017; Zarei et al., 2012).

Pain is a sensory response to unpleasant experience associated with actual or potential damage to tissues either by internal or external stimuli (Amanat, 2004). Pain can be generated by mechanical trauma such as insertion of dental needle, distribution of analgesia, or removal of dental needle from tissues. Patients' stress and the secondary innervation of the anatomical variations are among the various factors that affect the maintenance of effective anesthesia (Wang et al., 2014). Despite its high success rate, the anterior maxillary anesthesia infiltration injection technique is associated with unpleasant feeling in the patients due to induction of intense pain upon insertion of the needle (Steele et al., 2013). The infiltration technique is an ideal method for maxillary injections (Garcia‐Godoy, 1982). Although this technique can be carried out quite easily, it does not desensitize the lips and the tongue and the duration of anesthesia is short. The infiltration technique allows the dentist to restore the patients' teeth on both sides of their jaw in a single session. Consequently, the stress associated with dental treatment can be considerable reduced due to reduction in the number of dental appointments. Other measures are also on research to decrease the injection pain which can be achieved by changing the gauge and size of the needle tips, changing the type of analgesia, and changing the speed of distribution of this substance in dental tissues (Wang et al., 2014). On the other hand, the new techniques such as warming and buffering the analgesia (Aravena et al., 2018; Davoudi et al., 2016) and application of the novel computer‐aided techniques for injection (Davoudi et al., 2016) were recommended for minimizing pain. In some cases, a local anesthetic gel is applied to reduce pain during insertion of the needle (Cho et al., 2017). In this regard, the needle tip bevel design considerably influenced the penetration of the needle into tissues and the reduction of pain and stress (Davoudi et al., 2016; Steele et al., 2013; Wang et al., 2014). Thus, carrying out a successful dental treatment in the maxillary region requires observing a number of factors that can reduce injection pain (Davoudi et al., 2016).

**Figure 1:**
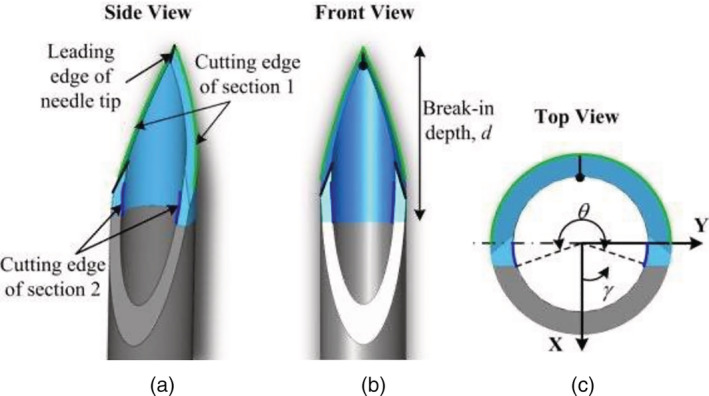
Shape of Transcodent needle

In comparison to the regular needle tips, the modern shapes and designs of the bevel, the three‐edge lancet grinding type in the Transcodent systems (Figure 1), has been claimed to offer better results. In this design, due to the sharpness of the needle tip and stiffness of the cannula silicone, the needle tip softly inserts in dental tissues resulting in patients' comfort and reduction of pain (Transcodent Co. Catalogue: 2020).

The present research aimed at determining the perceived pain upon the anterior maxillary infiltration injection using Transcodent painless needle (27G, short) tips in comparison to the regular needle tips.

## 2 MATERIALS AND METHODS

A statistical power analysis was performed for sample size estimation, based on effect size of a pilot study. With *α* = 0.05 and test power of 80%, the projected sample size needed with the estimated effect size was approximately 22 cases. To escalate the power of the study it was increased to 30 cases.

In the present research, a split‐mouth and randomized triple‐blind clinical trial was carried out among 30 patients who were candidates for cosmetic treatments in the Restorative Department of the university and volunteer to participate in the research and signed the Informed Consent form. The design of the study was approved in the university Ethics Committee of Vice Chancellor for Research (NO: REC.1398.079).

The study participants did not suffer from any systemic disease as determined by a written health history and oral questioning, they did not take any medication in the 12 h prior to the procedure that could change pain perception, and they all needed maxillary canine injection on both sides together with the completely sound canine teeth. The exclusion criteria were as follow: under 18 years old and over 60 years old, being pregnant, being allergic to local anesthetics and sulfites, active sites of pathosis in the area of injection, or inability to give informed consent.

Each patient received two similar supraperiosteal injections in the labial vestibule and close to the root apexes of the maxillary canine teeth. The injection was carried out through infiltration of Lidocaine HCL 2% with Epinephrine 1:100,000 (Persocaine, Daroupakhsh, Tehran, Iran) on both sides using two types of short needle tips: Transcodent painless needle tip 27G (Transcodent GmbH &Co., Sulzer (n.d.), Switzerland) and regular needle tip 27G (Soha Co., Tehran, Iran). The same dental specialist operated the injection on both sides so that the quality of both injections were similar and at a good level without any difference, Operator and patients were unaware of the overall aim of the study to minimize bias. Moreover, the injection pain was evaluate by an individual blind to the type of needles and statistical analyst was unaware of patients' grouping and interventions. The canine teeth were chosen on both sides in order to decrease the bias resulting from the distribution of the anesthetic medication from midline after the first injection, and both cartridges were kept at room temperature (approximately 22°C, 72°F; Malamed, 2019). Before injection, Benzocaine 20% topical gel (Topex, Sultan Dental Products, Hackensack, NJ) was administered on labial vestibule of both regions for 2 min to achieve topical anesthesia (Cho et al., 2017). Both injections were carried out in the mucobuccal fold above the apex of each tooth (Malamed, 2019). Before the infiltration injection, each subject was instructed on how to rate the pain for needle penetration and deposition of anesthetic solution by using a 100 mm Heft‐Parker Visual Analogue Scale (VAS). Immediately after the injection of 0.9 mL Lidocaine solution within 30 s in the site, level of pain was measured and recorded by the patients. Five minutes after the first injection, the site of injection was stimulated by a cotton roll to confirm the successful anesthesia and ensuring that no pain was felt. Then, the injection by another type of needle was performed on the opposite side and VAS form was filled out again by the patient. The sequence of injection for each patient was randomized by a balanced block random method using Excel software to determine the order of the needles and which side was injected first.

Taking into account the dependent nature of the data and following a normal distribution, the statistical analysis was carried out using the parametric paired *t*‐test. A 5% significance level was considered (*α* = 0.05) for statistically significant difference between two means.

## 3 RESULTS

Thirty volunteers participated in this study. There were 17 women and 13 men with a mean age of 27.3 ± 6.8 years. The level of pain (VAS) in the patients was estimated immediately after the anterior maxillary infiltration injection using Transcodent painless needle tip. The mean standard deviation of this pain was 18.3 ± 10.7 mm while that using regular needle tips was over twofold higher (43.1 ± 13.1 mm).

The mean and standard deviation of the pain level discrepancy VAS in the two groups was estimated as 25.1 ± 11.6 mm, and the standard error of pain level discrepancy in both groups was the same (2.1 mm). According to the results of the paired *t*‐test, there were significant discrepancies with respect to the pain level VAS in both groups (*p* < 0.001).

## 4 DISCUSSION

In the present research, the patients' pain level VAS scores in the anterior maxillary infiltration injection were studied by comparing the Transcodent painless needle tip with regular needle tips. In accordance with the results, using Transcodent painless needle tips have a significant impact on reducing the level of pain in the patients when compared to the injection with the regular needle tips. The patients' mean pain level during the anterior maxillary infiltration injection was over twofold lower when using Transcodent painless needle tip than the regular needle tips. Due to the modern bevel design of the three‐edge lancet grinding type, the Transcodent system was claimed to improve patient's comfort and increase softness of the injections. Consequently, the patients' pain level was reduced during the injection of the analgesia. However, this claim largely by the manufacturers needed to be independently investigated.

The Lancet type of cutting edges plays a crucial role in the injection needle tips. In this design, the performance of the needle tip is improved due to the concentration on the needle–tissue interface in lancet at the first moments of tissue cuts. At the same time, two distinct phases were detected on the curve of the input forces of the needle tip during the injection: in the first phase, the tissue was deformed and the force was increased without cutting the tissue. In the second phase, the tissue was cut and the force was suddenly decreased (Abolhassani et al., 2007; Moore et al., 2011, 2012; Okamura et al., 2004). It seems that these properties of cuts in the lancet design are associated with the softness of the injections and consequently, the reduction of pain.

At the same time, a study on the subcutaneous injection using different gauges reported that there was no significant difference in the levels of pain in the various groups (Montgomery et al., 2005). However, it was reported that the levels of pain in VAS were affected by the needle tip bevel design (Omoigui et al., 2006). Given the study was conducted on merely 20 patients, however, more research were needed in this regard.

To the best of our knowledge, no study was conducted regarding the effects of using Transcodent painless systems in comparison to the regular needle tip on the intensity of the pain level in humans. Nevertheless, some studies were carried out concerning the effects of various needle tip bevel designs on the results of the patients' pain. For instance, Dau et al. investigated the effects of the needle tip design on the patients' pain perception. The designs examined in the study included scalpel, three‐edge lancet grinding bevel, and regular bevel needle. Based on the results, the lower injection pain level was observed in the scalpel group in comparison to the three‐edge lancet grinding bevel and regular bevel needle. Also, injection with the scalpel design needle tips was associated with lower pain levels (Dau et al., 2017). This research indicated the importance of the needle tip bevel design on reducing the patients' pain. Furthermore, McPherson et al., studied the effects of using dental local anesthesia injection needle in both standard and larger sizes (from the same gauge) on reducing the injection pain of nerve block of long buccal and inferior alveolar nerve. They found no significant difference between using a 27‐gauge large needle and a 27‐gauge standard needle on the reduction of pain (McPherson et al., 2015).

Wang et al., carried out a study on the proper bevel design of the needle tips on the reduction of the internal forces and the length of the bevel. The study demonstrated that in the needle tips with Lancent bevel, insertion forces were 11% lower and the length of the bevel was 46% shorter than the commercial samples used from the same rake angle (Wang et al., 2014).

Candiotti et al., investigated the influence of the position of the needle tip bevel (bevel up and bevel down) on the intensity of pain pertinent to the subcutaneous injection of Lidocaine 1%. The higher levels of pain were reported by the patients in the bevel down position when compared to the bevel up position 2009. Besides, in a study conducted by Omoigui et al., the result of using needles with dual bevel and bevel with a lower angle in the subcutaneous injection was investigated. The results revealed that a lower level of pain was reported in the group with the needles with a low angle bevel design (Omoigui et al., 2006).

The results of the above‐mentioned studies demonstrated the importance of the needle tip bevel design on injection pain in patients. The results of the present study also indicated that using bevel design such as three‐edge lancet grinding was important in reducing injection pain in patients and resulted in a significant reduction of their pain when compared to the regular needle tips.

Previous studies estimated the effects of different needle tip designs or other parameters on patients' pain level: Once in an experimental group and once in a separate control group. The subsequent phases of measuring the levels of pain in these research were carried out 2 weeks after the first measurements and were called the wash‐out period (Fukayama et al., 2002; Parirokh et al., 2012). The results of such studies might not be reliable due to the fact that perception of pain is influenced by the physical, mental, and emotional status and varies on different days. In addition, in the protocols based on the measurement of levels of pain in various sessions, there is a possibility of error considering that specific standards of a person might not be stable in the perception of pain. On the other hand, in individuals with high anxiety, different levels of pain intensity are reported in the session immediately after the treatment and remembering the same pain in the subsequent sessions (Kent, 1984, 1985). In order to remove these errors, infiltration injection of the anterior maxillary regions was performed in the present study with two types of needles in the same session, and the patients were requested to rate the level of pain on VAS immediately after the injection. Considering the moment of the needle insertion and the first seconds after injection being the most painful moments in anesthetic infiltration, estimating the pain immediately after injection can show a higher VAS score (Cho et al., 2017).

On the other hand, in the research pertinent to the measurement of injection‐related pain, all conditions should be assessed and controlled. The injection condition, operator's characteristics, and the speed of injection should be standard and their changes on individuals should be equal to zero or very little in order to prevent the probable errors.

Due to the painful injection in the maxillary anterior region, it is highly recommended to use topical anesthesia in the mucobuccal fold before needle insertion. Moreover, highly anxious patients reported higher pain scores. Therefore, using topical anesthetics reduced the effect of anxiety on perceived pain. It was demonstrated that topical anesthetic application significantly reduced both insertion‐ and injection‐related pain during infiltration anesthesia in the maxilla (Cho et al., 2017). Therefore, in the present study topical anesthetic gel used before infiltration on local anesthesia in both sides.

To statistically analyze the level of pain in the local anesthetic injection and placebo groups, previous studies employed independent tests such as Mann–Whitney U Test or Independent *t*‐test (Drum et al., 2011; Fukayama et al., 2002), while some used paired *t*‐tests or McNemar tests (Bhalla et al., 2009; Hersh et al., 1996; Parirokh et al., 2012). In the independent tests, an extra variable is included in the test, which is the result of the independence of the observations. In other words, pain variables are measured and reported in two different cases. In the present study, the paired *t*‐test was employed for statistical comparisons, which is not affected by this change. Paired observations depend on each other in an individual and the same person is used for measurement of the intensity of pain. On the other hand, the paired test does not require two group samples to have equal variances (as opposed to the independent tests). Thus, the paired test has a higher statistical strength than an independent test and these tests are appropriate for the paired protocols, in which the local anesthesia group and placebo group are assessed on similar cases (Rice, 2006).

Besides treatment‐related factors, mental variables such as fear and anxiety also affect pain intensity. It was found that patients' anxiety leads to an increase in the duration and intensity of pain (Johnson & Primosch, 2003), which should be considered in the studies pertinent to patients' injection‐related pain by various methods or different designs of needle tips.

The pain experienced during local anesthesia injection comprises of two parts. The first is the feeling of pain upon insertions of a needle, which is short‐term pain. The secondary pain is felt upon the activation of pain recipients that reacts against chemical factors in infiltration and the damage to the tissue. The secondary feeling of pain is more intense and lasts longer (Zilinsky et al., 2005). Taking into consideration that infiltration injection in the anterior maxillary region and IAN block injection in the mandible are among the most painful and most applicable injections in the mouth region, the present research limited the study to the anterior maxilla to decrease study bias. On the other hand, precise insertion of the needle and slow distribution of the analgesia in the tissue can reduce needle insertion pain (Friedman & Hochman, 1997). In the study by Primosch and Brooks, it was revealed that the slow distribution of the anesthetic substance can lead to a significant reduction of the level of pain in comparison to its fast distribution (Primosch & Brooks, 2002). On the other hand, the experience of pain during injection of the anesthetic substance as an unpleasant experience can be affected by a variety of biological, mental, and social factors such as fear, anxiety, and previous experiences of injection of the anesthetic substance (McGrath, 1985). Previous experience of pain, an individual's expectation of pain, and cultural differences can also influence the intensity of pain in patients. Since previous painful experiences from local anesthesia injection can increase the person's anxiety, measuring the amount of pain in these patients can be challenging. At the same time, the intensity of pain is an individual‐specific subject, thus, the same qualitative and biological parameters (e.g., speed of the local anesthetic substance injection) can result in different experiences of pain in the patients (McPherson et al., 2015). Besides, from the clinical point of view, people with lower levels of anxiety can be included in research for the purpose of improving the reliability of pain measurement (Nusstein et al., 2004).

Different criteria were employed for the measurement of pain after insertion of the needle or the injection pain. Pain is a subjective phenomenon and its intensity can be affected by various physical and mental factors. Thus, patients' anxiety should be measured and determined prior to interventions. Furthermore, pain estimation, unlike many other clinical experiments, is an experimental process with many variables that can affect the outcome. With correct design and precise explanation of the VAS criteria to the patients, this method can be a valid technique in pain estimation (Price et al., 1983).

One possible limitation of this study is that patients' levels of pain should be measured immediately after the infiltration injection of the anterior maxilla. It seems that in case of separation of the levels of needle insertion related to pain from the levels of pain caused by the anesthetic solution injection, the precision of the results can be improved. On the other hand, it was claimed that the patients are incapable of differentiating these two types of pain (McPherson et al., 2015).

## 5 CONCLUSION

The results of this randomized, split‐mouth clinical study highlighted the potential use of Transcodent painless needle for reducing the injection pain of maxillary anterior infiltrations.

## CONFLICT OF INTEREST

The authors deny any conflicts of interest.

## AUTHOR CONTRIBUTIONS

**Farnoosh Razmara**: Conceptualization; data curation; formal analysis; investigation; methodology; project administration; supervision; validation; visualization; writing—review and editing. **Arjang Baghi**: conceptualization; data curation; formal analysis; funding acquisition; investigation; methodology; validation; writing original draft. **Farzaneh Afkhami**: conceptualization; investigation; methodology; project administration; supervision; writing—review and editing.

## ETHICS STATEMENT

This study was approved by the Ethics Committee of Tehran University of Medical Sciences.

## Data Availability

Data available on request from the authors.
